# Supplementary material for: A general mechanism of ribosome dimerization revealed by single-particle cryo-electron microscopy
Source: Nat Commun. 2017 Sep 28;8:722. doi: 10.1038/s41467-017-00718-x (PMC5620043; doi:10.1038/s41467-017-00718-x)
Supplement: Supplementary file 3 — Supplementary Information [file 41467_2017_718_MOESM3_ESM.pdf]

File Name: Supplementary Information
Description: Supplementary Figures and Supplementary References

File Name: Supplementary Movie 1: N-terminal domain of HPFlong
Description: The model of the N-terminal domain of HPF^{long} fitted into the experimental density

File Name: Supplementary Movie 2: C-terminal domain of HPFlong
Description: The model of the C-terminal domain of HPF^{long} fitted into the experimental density at the dimerisation interface

File Name: Peer Review File

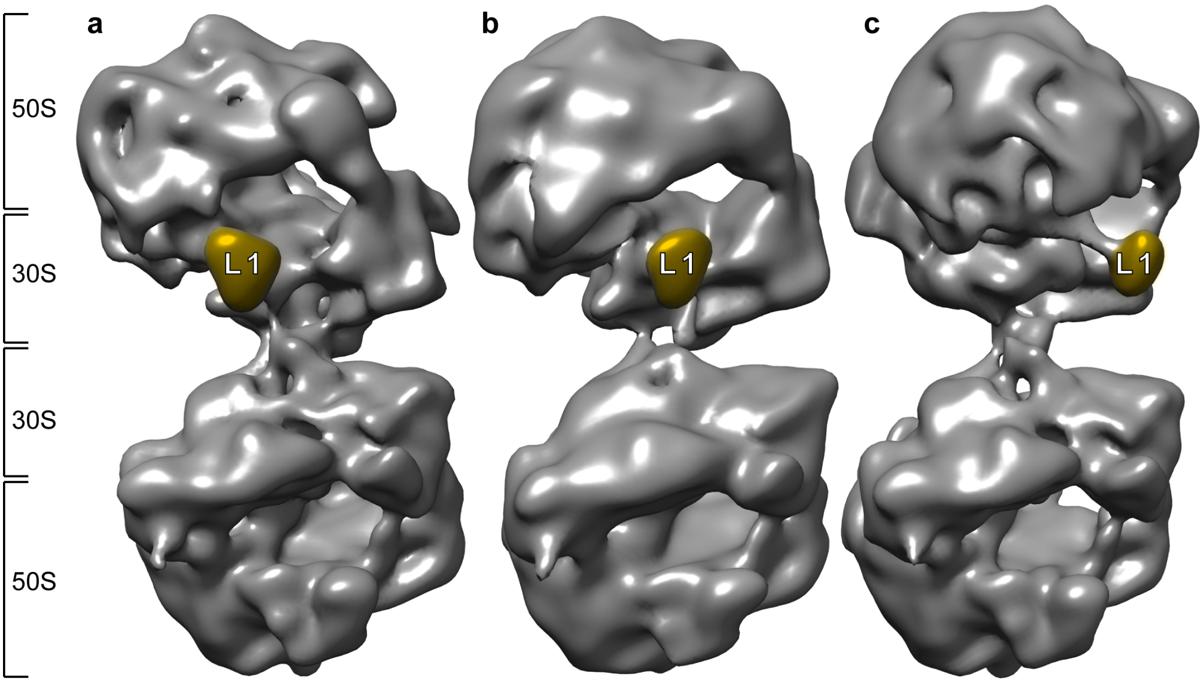

Supplementary Figure 1: Classification of 100S dimers. Three dimer conformations at a resolution of 19 Å (**a**), 22 Å (**b**) and 20 Å (**c**), respectively. They were aligned on the basis of the bottom 70S of class **a**. Classes **a** and **c** represent the widest range of rotational displacement (55°) between the two ribosomes. The extent of rotational freedom around the long axis can be seen by comparing the positions of the L1-stalk (gold) of the top ribosomes. L1 of the bottom ribosomes is not visible in this projection. The percentage of particles from the clean data in each conformation is 59 (**a**) 5 (**b**) and 35% (**c**).

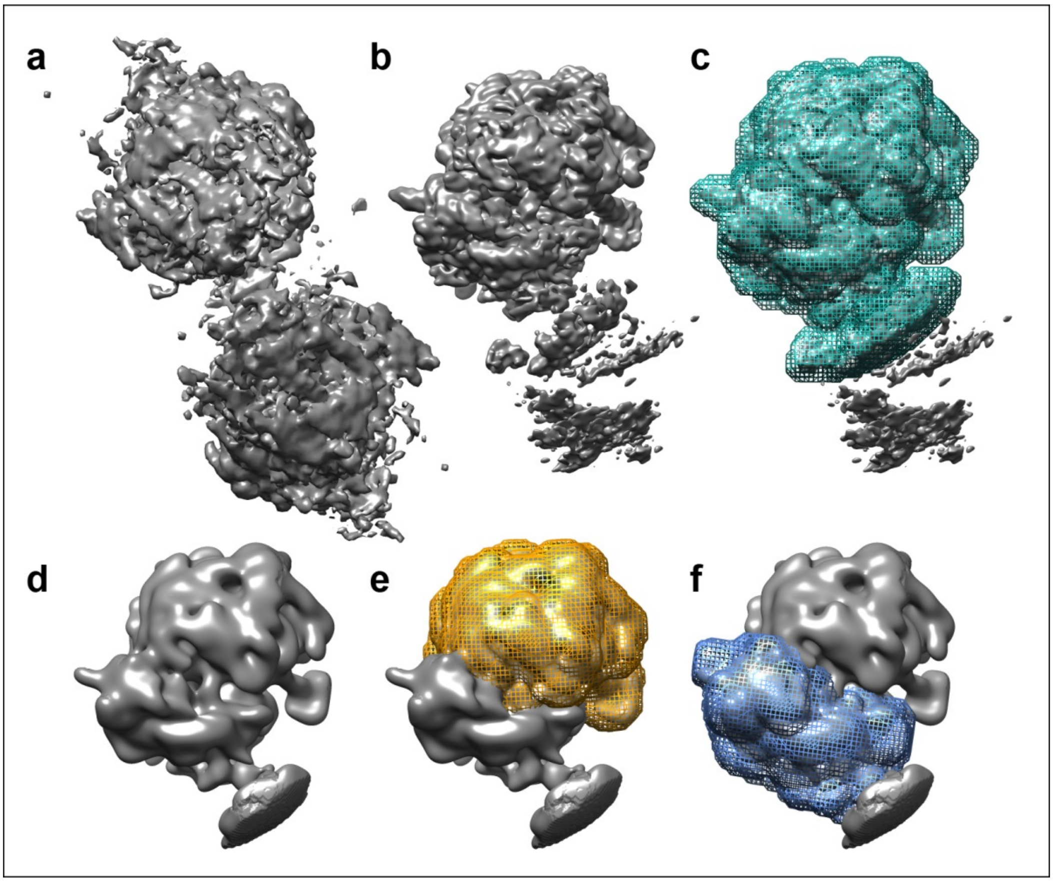

Supplementary Figure 2: The reference maps and masks used to obtain the final maps. **a**) Reference map that was made symmetrical for the first round of 3D classification of the combined dataset. **b**) Reference map and **c**) the monomer mask used to further classify the clean data (comprised of nine recombined, good classes) into three classes. Of these three, two were recombined to subsequently run refinement with the best class (**d**) as a starting model and a 50S mask (**e**). The same dataset was classified into three more classes with a 30S mask (**f**) and subsequently refined using the same reference (**b**) and mask (**f**). All reference maps were filtered to 60 Å to avoid a strong influence of the reference on the results.

After combining the two datasets (70S and 100S micrographs), one iteration of cleaning was performed. This was done with a 100S initial model that was artificially made C2-symmetrical to get equal weight in both halves (Supplementary Fig. 2a). Nine good 3D classes were obtained with resolutions below 30 Å. These were recombined and further classified into three classes, using the best class as reference (Supplementary Fig. 2b) and a mask. This mask was the difference between a circular mask to focus on one monomer and the mask that was created during post-processing (Supplementary Fig. 2c). Of these three classes, two were recombined, the best which was taken as a reference (Supplementary Fig. 2d), for refinement based on a tightly fitting 50S mask (Supplementary Fig. 2e). Meanwhile, that same dataset was also further classified into three classes with the 30S mask (Supplementary Fig. 2f). This yielded one good class that was refined using the same reference and mask. This map agreed with the result of the 50S mask, but did not improve resolution in the 30S and was not further used.

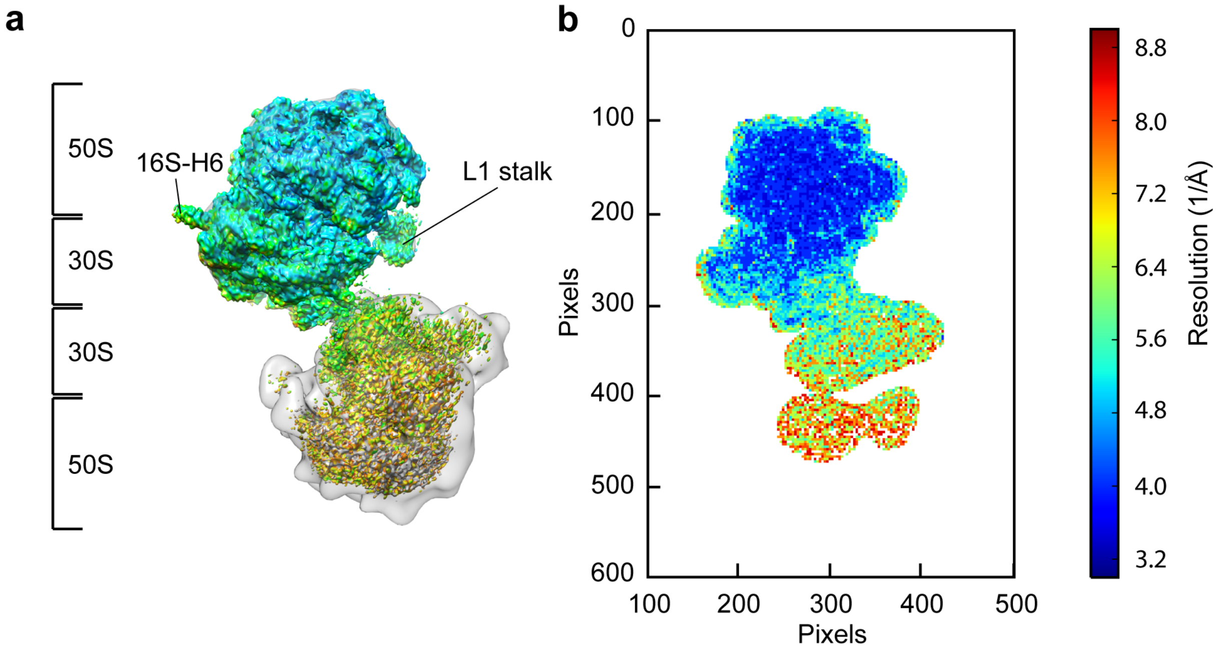

Supplementary Figure 3: Resolution of 100S ribosome EM map refined with a 50S mask. a) 5.6 Å resolution map colored by resolution distribution; the scale bar indicates the resolution in Å. For reference, the low-resolution map (as in Supplementary Fig. 1a) is shown in the background. **b**) Internal map resolution can be observed from a slice through the density of panel **a**. Further validation of the model in the form of 2D classes, angular distribution as well as the Fourier Shell Correlation curve are shown in Supplementary Figures 4 and 5.

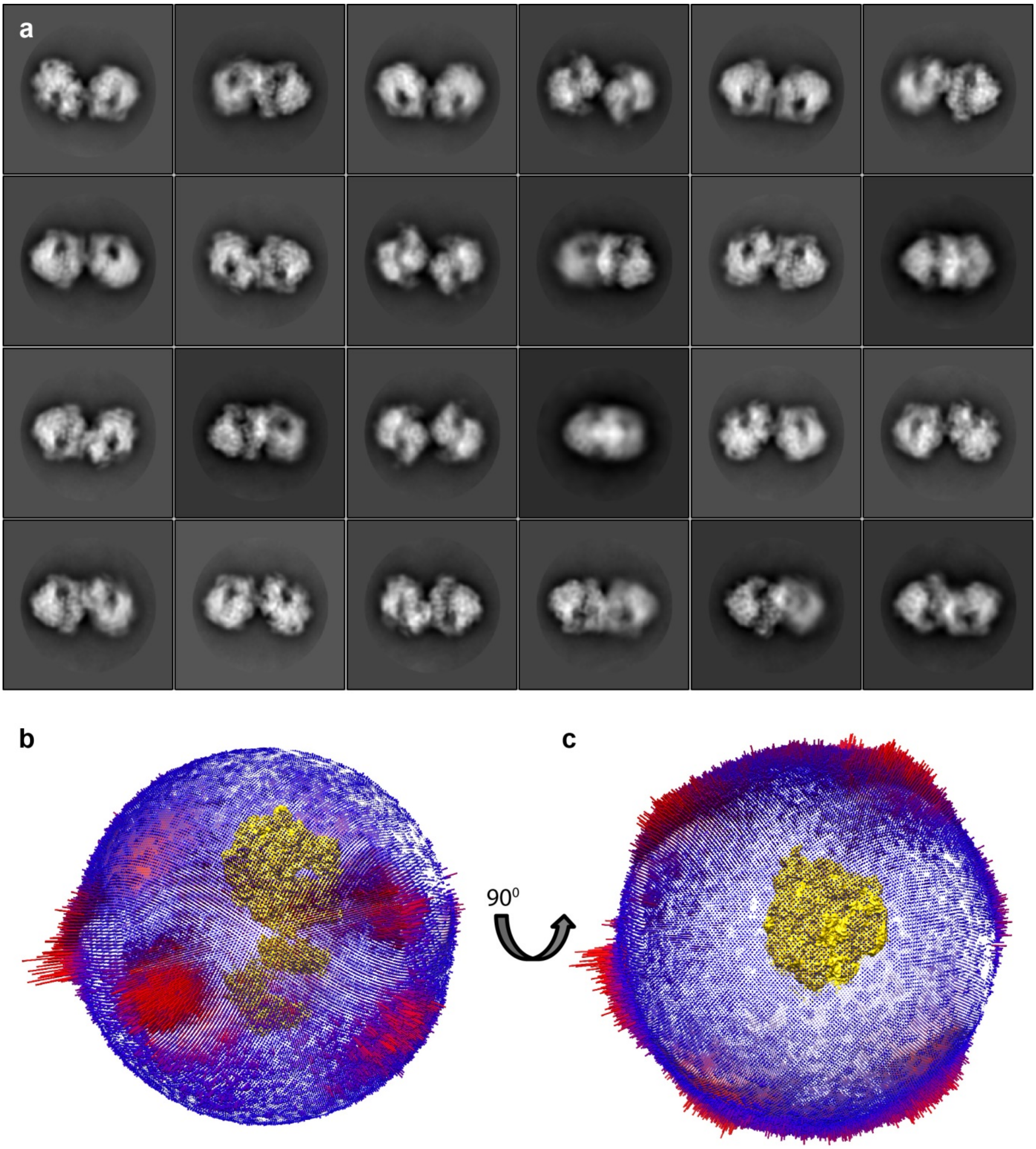

Supplementary Figure 4: Preferred orientations and angular distribution of the 43,530 particles that were used in the final 5.6 Å map (Supplementary Fig.3). a) 2D-classification of the final dataset. **b)** and **c)** Angular distribution of the different particles that were combined to make the final 5.6 Å map (gold). The different angles that are present in the data are represented by bars that are short and colored blue when the number of particles with that angle is low, and they are longer and more red when particles with that angle are more abundant. Although there is preferred orientation for specific side-views, there are no large patches of missing angles, and even the top-views are present in the data. Panel **c** is rotated 90° with respect to **b** to show the angles from the top-view.

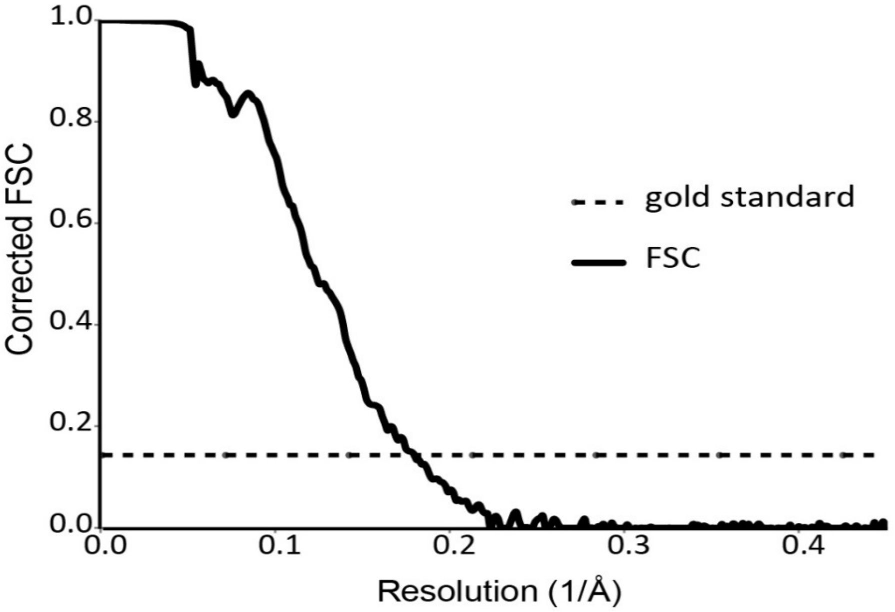

Supplementary Figure 5: The Fourier Shell Correlation (FSC) curve along with the gold standard criterion[1]. The sharp dip at 18.9 Å (1/Å 0.05) was removed, because it is an artifact attributed to the application of a mask[2].

16S rRNA

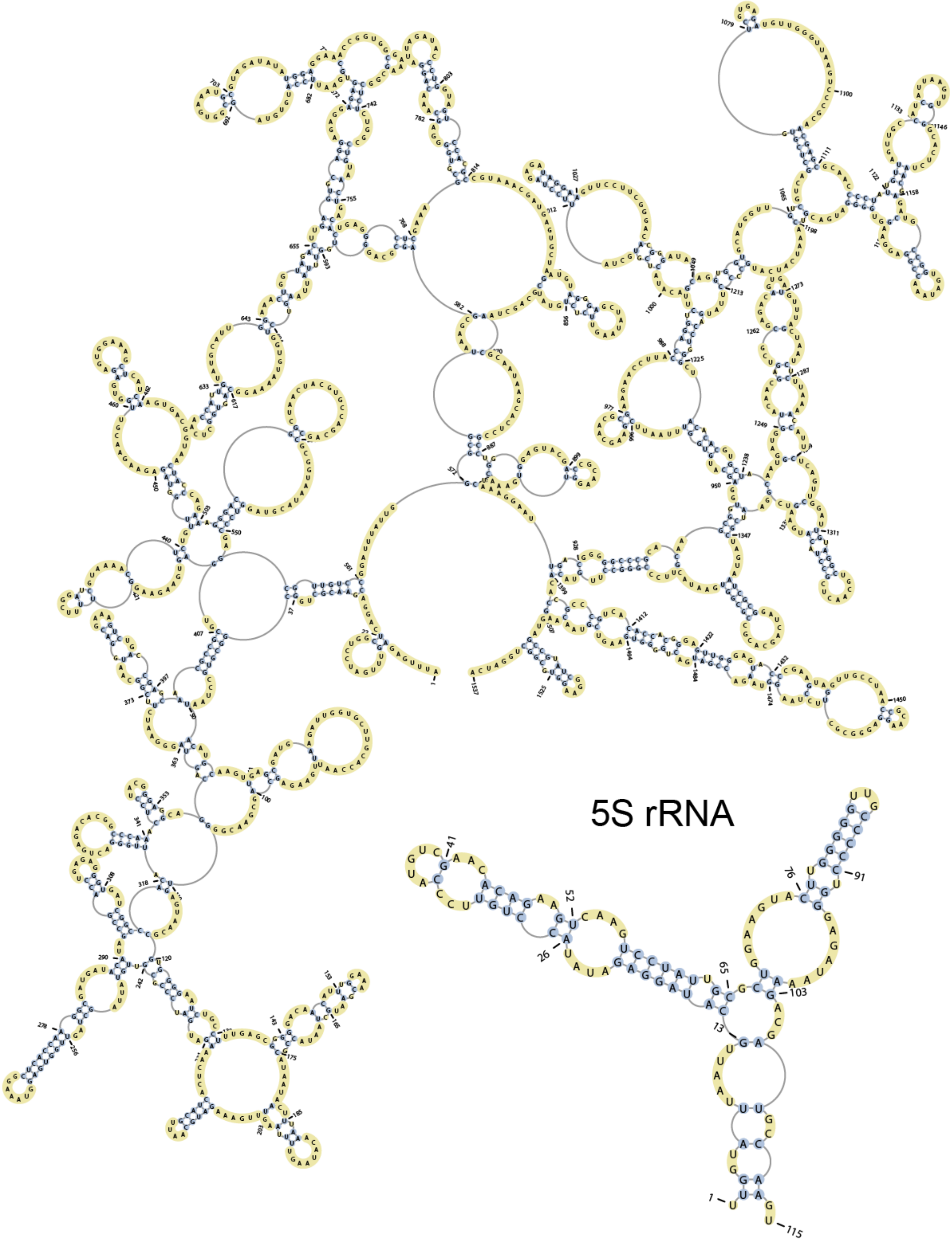

23S rRNA (A)

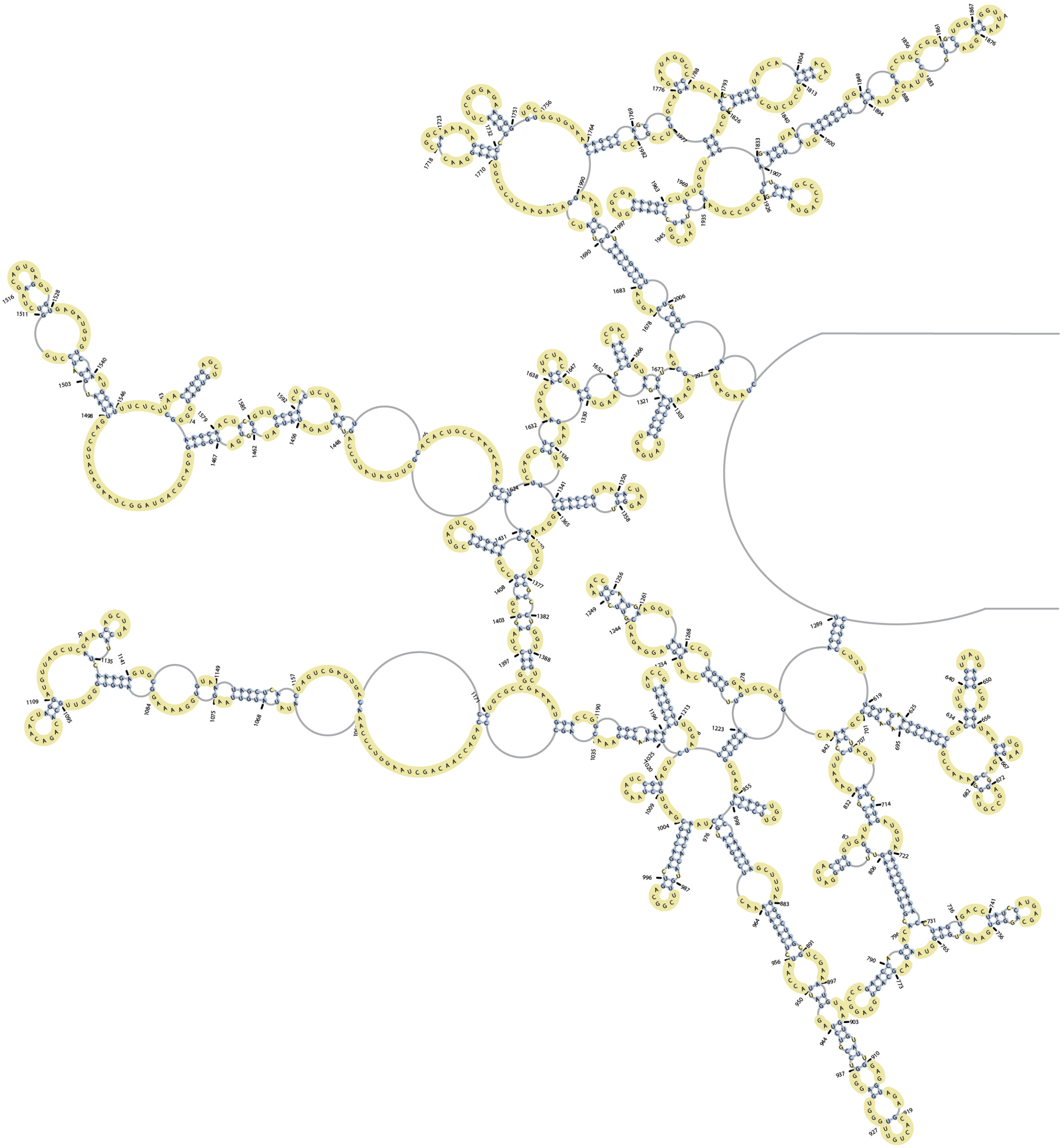

23S rRNA (B)

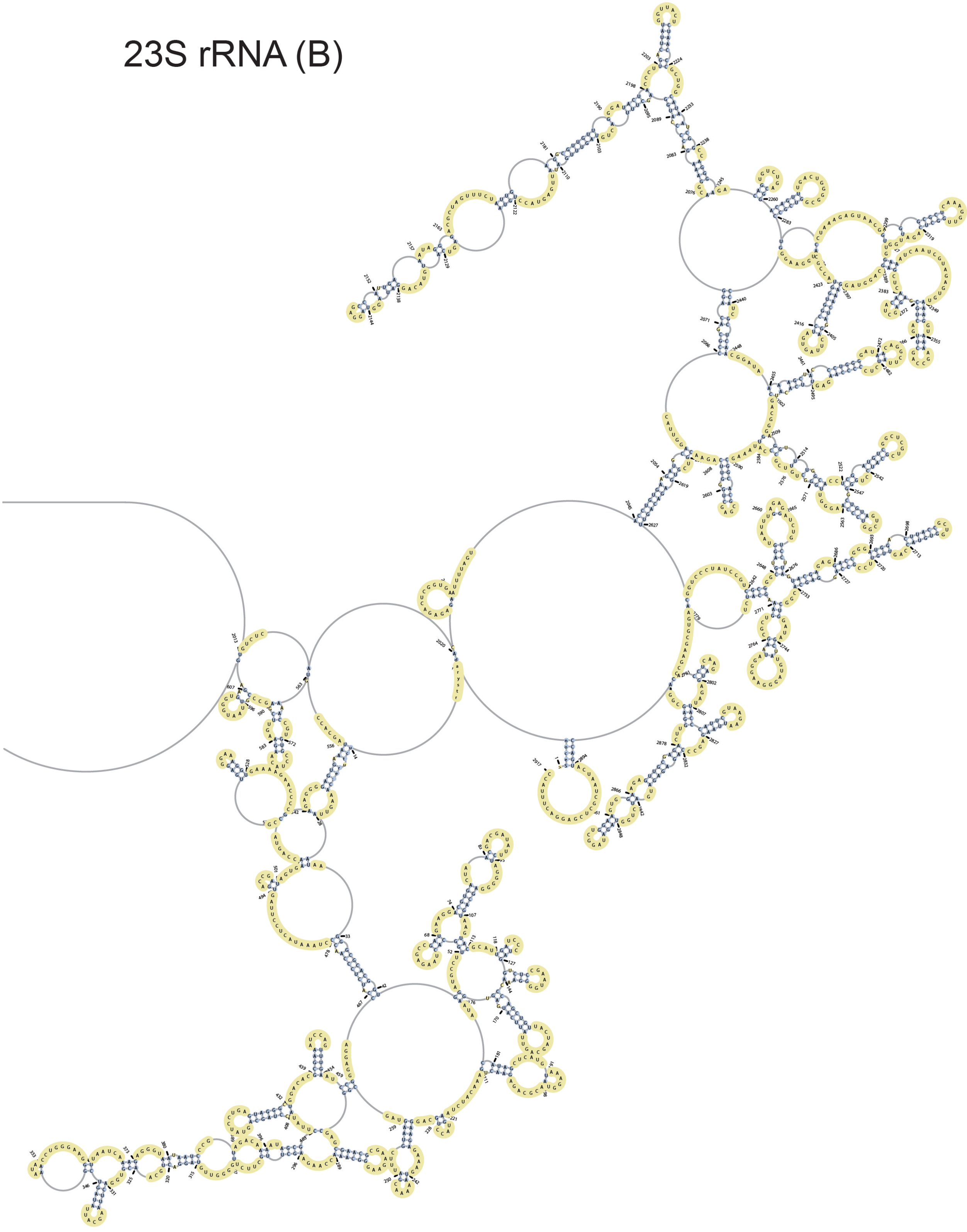

Supplementary Figure 6: Secondary structures of the 16S, 5S and 23S rRNAs from *L. lactis*. The rRNA is highly conserved between *L. lactis* and *B. subtilis*. The main deletions and insertions compared to *B. subtilis* are located in the positions of H10, H17 in 16S and H9, H18, H38, H54, H57, H63, H68, and H98 in 23S from *L. lactis*.

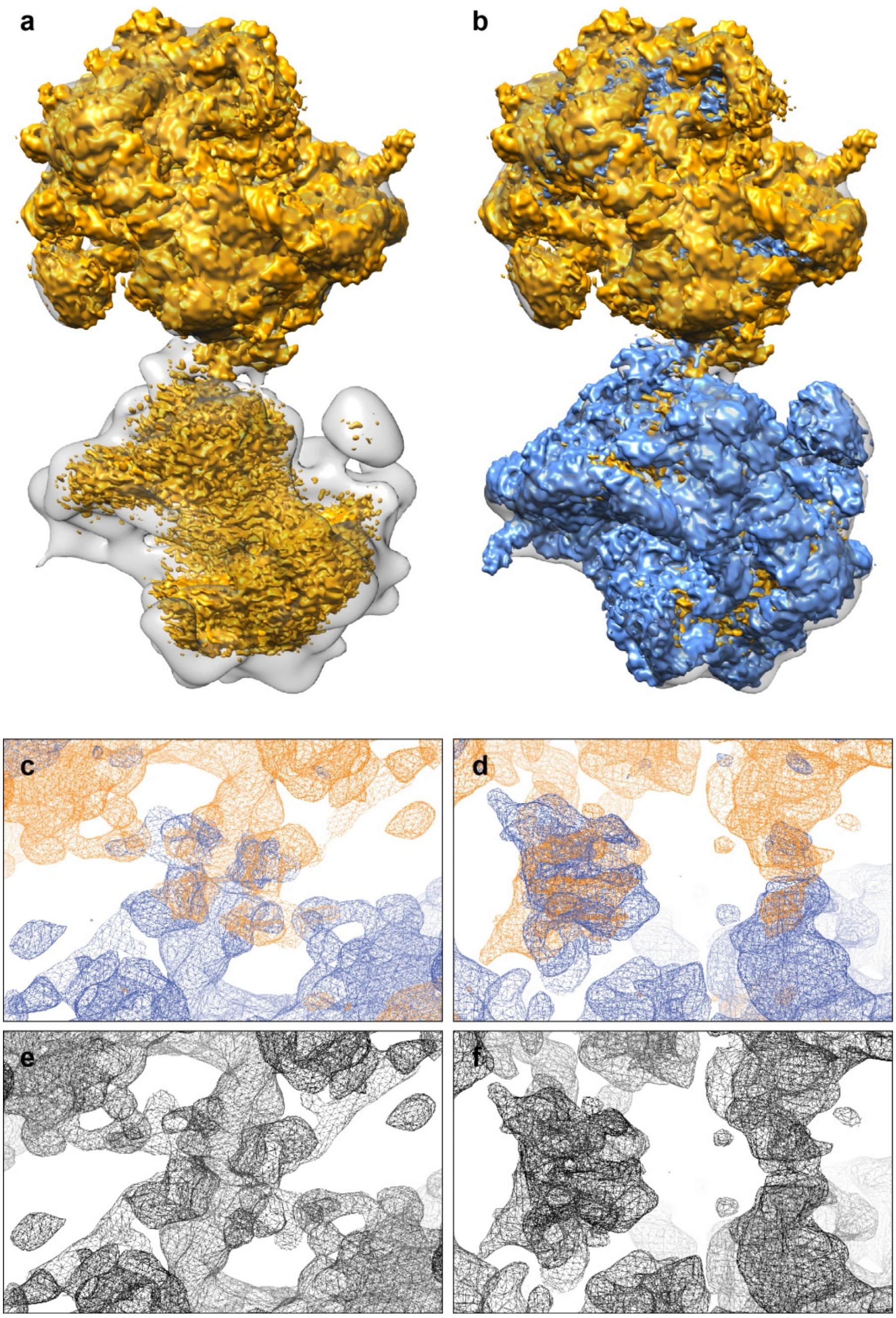

Supplementary Figure 7: Reconstruction of the 100S density map of the ribosome dimer from *L. lactis*. Two copies of the 5.6 Å map (blue and orange) were fitted in the 100S low-resolution structure (grey) (see Fig. 1a, main text) and subsequently into each other (**a** and **b**). Zooming in on the interface of the dimer along the symmetry axis (**c**) and rotating by 90° (**d**) shows that the combined maps complement and improve the density at the interface. The single 100S map (**e** and **f**) that was used to refine the interface was created by taking the maximum voxel value between the two aligned maps, to obtain for each voxel the largest signal. The higher the value, the better the signal-to-noise ratio, and thus this value is more likely to be accurate than the lower value from the other map. There is high correlation in the density between the two maps for the area that corresponds to the C-terminal domains of HPF^{long}. No distortions or other artifacts were observed in the region of the map corresponding to the well-known structure of protein uS2, which is also located at the interface. Furthermore, the obtained density for the C-terminal domain of HPF^{long} is in accordance with the structures and volumes of the homology models (PDB-codes 3KA5 and 3K2T).

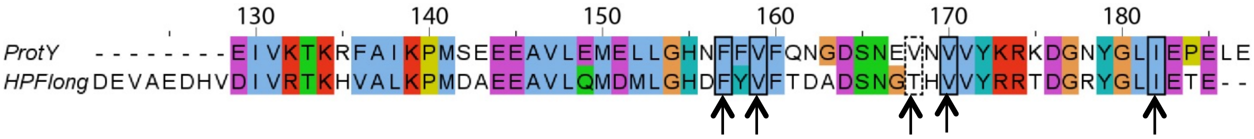

Supplementary Figure 8: Sequence alignment in CLUSTAL O [3] of the C-terminal domain (residues 121-185) of HPF^{long} from *L. lactis* and the ribosome-associated protein Y (PSrp-1) (PDB-code 3KA5) from *Clostridium acetobutylicum*. Highlighted are the residues at the dimer interface; four out of five interface residues (indicated by arrows) are preserved between the two species.

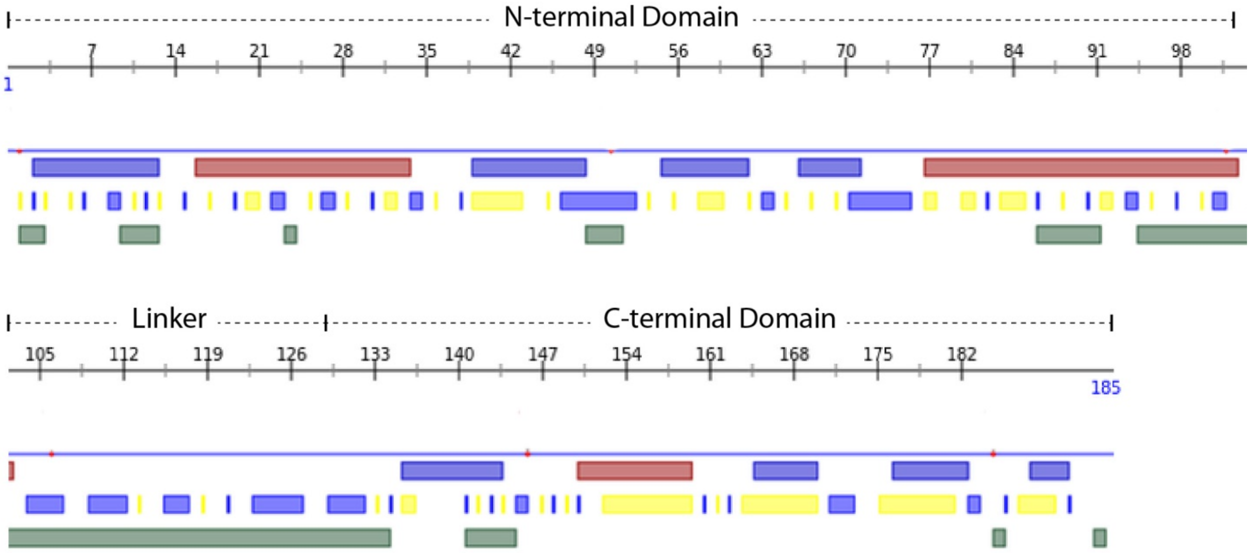

Supplementary Figure 9: Structure prediction of HPF^{long} from *L. lactis*, using the Predict Protein server [4]. To predict the structure, the server aligned a total of 32 homologous sequences. The residue numbers of HPF^{long} from *L. lactis* are indicated above the line. The bars underneath the line indicate the predicted secondary structure: β-strands (blue bars) and α-helices (red bars), the solvent exposure in blue (exposed) and yellow (buried), and the prediction of disordered areas in pale green[5].

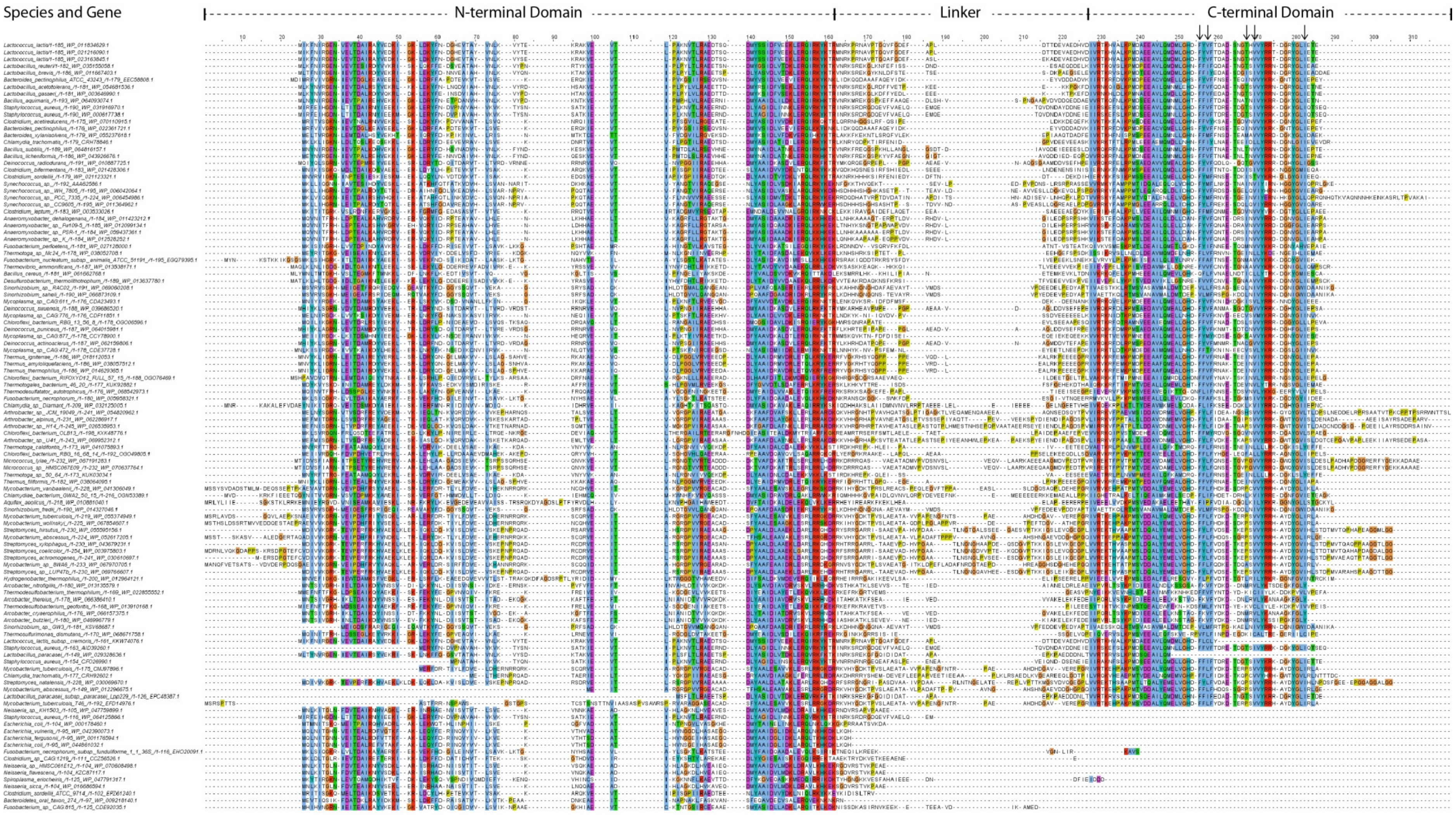

Supplementary Figure 10: Sequence alignment of *L. lactis* HPF^{long} homologues. Arrows indicate the residues that are involved in the hydrophobic interactions of the HPF^{long} dimer interface.

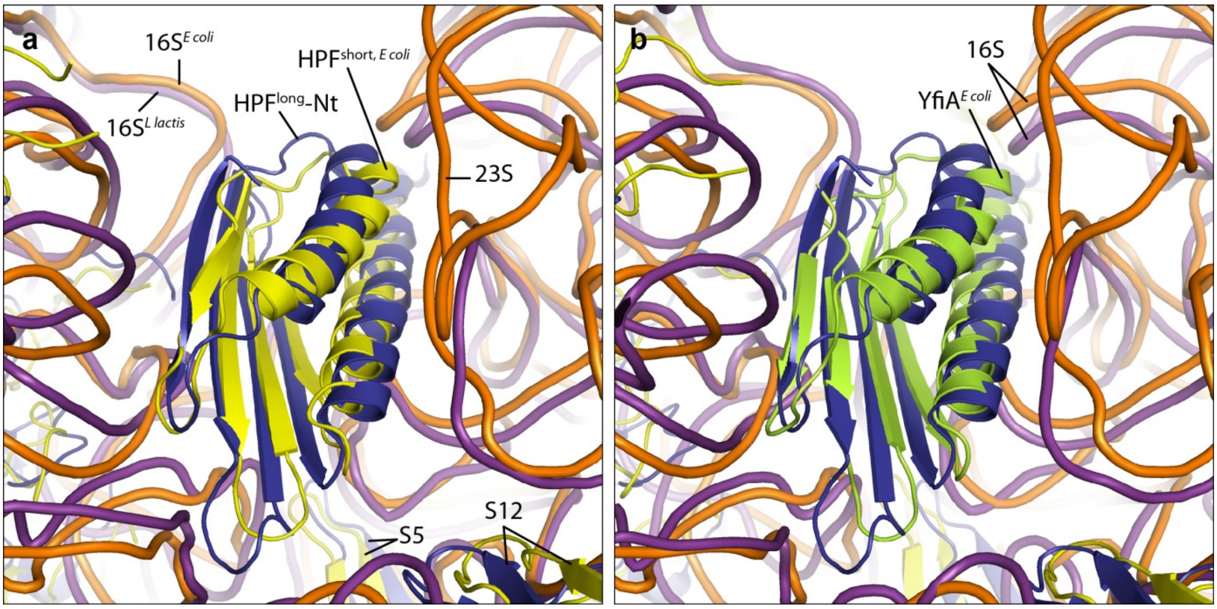

Supplementary Figure 11: Superposition of structures of HPF^{long} N-terminal domain in *L. lactis* and HPF^{short} and YfiA in *E. coli.* **a**) Superposition of HPF^{short} (yellow) in *E. coli* and HPF^{long} N-terminal domain (blue) in *L. lactis*. HPF^{long}, HPF^{short} and YfiA mainly interact with the 16S rRNA (*L. lactis*: orange; *E. coli*: purple). **b**) Superposition of YfiA (green) and HPF^{long} N-terminal domain.

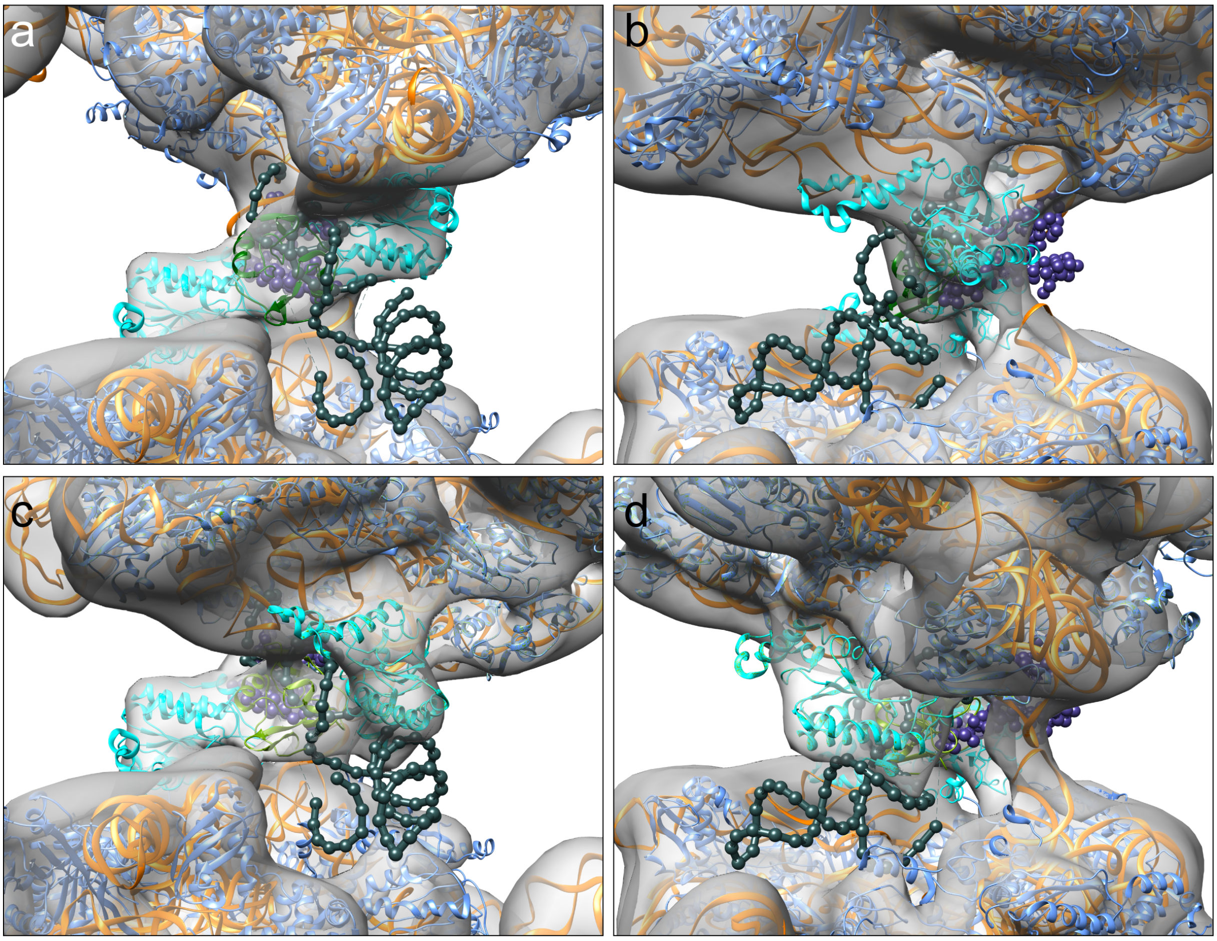

Supplementary Figure 12: Superposition of a pre-initiation complex on the open and closed states of the *L. lactis* ribosome dimers. The locked pre-initiation complex of *rpsO* mRNA (grey ball-chain) - uS15 (purple ball-chain) with an *E. coli* ribosome[6] (not shown) superimposed on the open (**a** and **b**) and closed (**c** and **d**) conformations of *L. lactis* dimers, illustrating how the interface of the dimer clashes with *rpsO* mRNA-uS15; 16S rRNA (orange), proteins (blue), uS2 (cyan) and HPF^{long} (dark green).

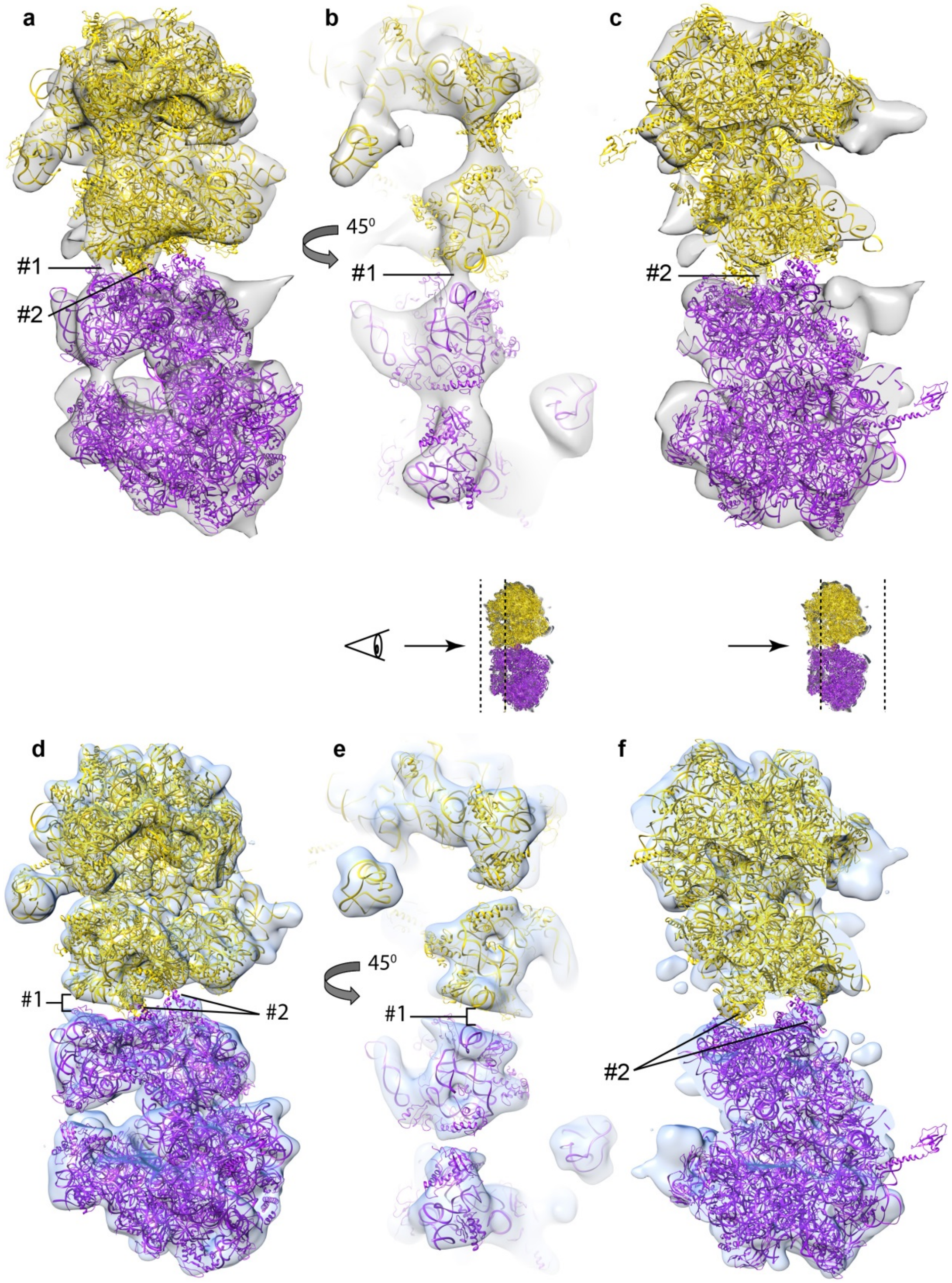

Supplementary Figure 13: Dimerization of *E. coli* ribosomes. Two copies (yellow and purple) of the structure of *T. thermophilus* ribosomes with *E. coli* RMF (PDB-code 4V8G) were rigid-body fitted into the EM maps of Ortiz *et al.*[7] (**a-c**) and Kato *et al.*[8](**d-f**). The map from Kato *et al.* was created by fitting two copies of the map, one rotated 180° with respect to the other, into each other to complete the 100S structure and taking the maximum voxel value between the two maps (as in Supplementary Fig. 7). The maps in panels **a** and **d** (rotated 45° with respect to **b**, **c**, **e** and **f**) demonstrate the general model-to-map fits. **b**, **c**, **e** and **f**) Cropped views, indicated between the dotted lines in the small insets. **b** and **e**) Interaction-site one (#1). **c)** and **f**) Interaction-site two (#2) as described in Polikanov *et al.*[9]. As can be seen when panel **b** is compared to **e**, both maps do not agree with respect to interaction-site #1.

Supplementary references

1. Scheres, S. H. W. & Chen, S. Prevention of overfitting in cryo-EM structure determination. *Nature Methods* **9**, 853-854 (2012).

2. Baker, M. L. *et al*. Validated near-atomic resolution structure of bacteriophage epsilon15 derived from cryo-EM and modeling. *Proc. Natl. Acad. Sci. U. S. A.* **110**, 12301-12306 (2013).

3. Sievers, F. *et al*. Fast, scalable generation of high-quality protein multiple sequence alignments using Clustal Omega. *Molecular Systems Biology* **7**, 539 (2011).

4. Rost, B., Yachdav, G. & Liu, J. The PredictProtein server. *Nucleic Acids Res.* **32**, W321-W326 (2004).

5. Schlessinger, A., Punta, M., Yachdav, G., Kajan, L. & Rost, B. Improved Disorder Prediction by Combination of Orthogonal Approaches. *Plos One* **4**, e4433 (2009).

6. Marzi, S. *et al*. Structured mRNAs regulate translation initiation by binding to the platform of the ribosome. *Cell* **130**, 1019-1031 (2007).

7. Ortiz, J. O. *et al*. Structure of hibernating ribosomes studied by cryoelectron tomography in vitro and in situ. *J. Cell Biol.* **190**, 613-621 (2010).

8. Kato, T. *et al*. Structure of the 100S Ribosome in the Hibernation Stage Revealed by Electron Cryomicroscopy. *Structure* **18**, 719-724 (2010).

9. Polikanov, Y. S., Blaha, G. M. & Steitz, T. A. How Hibernation Factors RMF, HPF, and YfiA Turn Off Protein Synthesis. *Science* **336**, 915-918 (2012).
